# Postprandial Levels of Branch Chained and Aromatic Amino Acids Associate with Fasting Glycaemia

**DOI:** 10.1155/2016/8576730

**Published:** 2016-05-05

**Authors:** Filip Ottosson, Ulrika Ericson, Peter Almgren, Jeanette Nilsson, Martin Magnusson, Céline Fernandez, Olle Melander

**Affiliations:** ^1^Department of Clinical Sciences, Lund University, Jan Waldenströms Gata 35, 21421 Malmö, Sweden; ^2^Department of Cardiology, Skåne University Hospital, Entrance 35, 20502 Malmö, Sweden; ^3^Department of Internal Medicine, Skåne University Hospital, Ruth Lundskogs Gata 3, 20502 Malmö, Sweden

## Abstract

High fasting plasma concentrations of isoleucine, phenylalanine, and tyrosine have been associated with increased risk of hyperglycaemia and incidence of type 2 diabetes. Whether these associations are diet or metabolism driven is unknown. We examined how the dietary protein source affects the postprandial circulating profile of these three diabetes associated amino acids (DMAAs) and tested whether the postprandial DMAA profiles are associated with fasting glycaemia. We used a crossover design with twenty-one healthy individuals and four different isocaloric test meals, containing proteins from different dietary sources (dairy, fish, meat, and plants). Analysis of the postprandial DMAAs concentrations was performed using targeted mass spectrometry. A DMAA score was defined as the sum of all the three amino acid concentrations. The postprandial area under the curve (AUC) of all the three amino acids and the DMAA score was significantly greater after intake of the meal with dairy protein compared to intake of the three other meals. The postprandial AUC for the DMAA score and all the three amino acids strongly associated with fasting glucose level and insulin resistance. This indicates the importance of the postprandial kinetics and metabolism of DMAAs in understanding the overall association between DMAAs and glycaemia.

## 1. Background

High circulating concentrations of branched chain and aromatic amino acids (BCAAs and AAAs) are associated with several characteristics of diabetes, such as increased glycaemia and insulin resistance [1–6]. In prospective studies, baseline systemic levels of BCAAs and AAAs have predicted future development of both diabetes and cardiovascular disease [5, 7, 8]. In particular a score of isoleucine (Ile), tyrosine (Tyr), and phenylalanine (Phe) has been a strong predictor [5, 7]. Whether these diabetes associated amino acids (DMAA) are causally related to insulin resistance and diabetes or just biomarkers for the underlying processes is still under debate and several possible mechanisms for causality have been discussed [9, 10]. Interestingly, high consumption of BCAA has also been shown to have positive health effects in terms of reduced risk of overweight [11]. The aforementioned studies all address the relation between diabetes and circulating concentrations of DMAAs in the fasted state. Although there is great potential in using these fasting metabolite measures as biomarkers for diabetes, they only provide information about a metabolic steady-state and leave us oblivious to the metabolic dynamics and flexibility of the subject. Several possible processes could contribute to increased circulating DMAAs, including protein dietary intake, altered DMAA uptake rate into tissue, breakdown of muscle proteins, and altered DMAA metabolism. Which of these processes, that predominantly causes the increase in fasting circulating DMAAs, has not yet been established? Assuming that the association between DMAAs and diabetes development is causal, it is instrumental to identify which of these principally different processes that explain the association, in order to finally establish whether changing the intake of DMAAs is meaningful for diabetes prevention.

Some studies have investigated the postprandial circulating levels of DMAAs, but they are performed in specialized settings, such as oral glucose tolerance test or after ingestion of a mixed meal [12, 13]. Recently, the postprandial plasma profile of DMAAs for morbidly obese subjects was shown to be altered after bariatric surgery, which simultaneously improved several risk markers for diabetes. After intervention, the amino acids showed a more rapid systemic appearance and clearance after a mixed meal and both pre- and postintervention postprandial profiles tracked closely to that of insulin. Fasting levels of DMAAs were also affected by the bariatric surgery but the effects were not as pronounced as in the postprandial state [14]. These results hint at the importance of measuring postprandial profiles of DMAAs and their potential involvement in the onset of insulin resistance and diabetes.

We hypothesized that the dietary protein source would differentially affect the postprandial plasma profile of DMAAs. Furthermore, assuming that circulating levels of DMAAs are not only regulated by the rate of muscle protein breakdown, we hypothesized that postprandial DMAA levels following a protein test meal would be more strongly associated with the diabetes prone phenotype of elevated fasting glucose than fasting DMAA levels. The aims were thus to test if 4 isocaloric test meals with different protein sources (dairy, fish, meat, and plant foods) would differentially affect the postprandial DMAA profiles. As elevation of fasting glucose is one of the most potent risk factors for future diabetes development we also aimed to examine the relation between both fasting and postprandial plasma concentrations of DMAAs and fasting glucose concentration. Last, as insulin resistance is a typical feature of the prediabetic phenotype, we also studied the relation between DMAAs levels and HOMA-index of insulin resistance.

## 2. Material and Methods

21 healthy unmedicated individuals (no history of metabolic or cardiovascular disease) in the age range of 21–62 years were recruited to the study through local advertisement. The participant's clinical characteristics are presented in Table 1. All participants provided informed consent and the study design was approved by the Ethics Committee of Lund University, Sweden.

### 2.1. Crossover Amino Acid Challenge

Participants made four separate visits in order to consume four different isocaloric test meals. All test meals contained 15 energy percent (E%) protein, 30 E% fat, and 55 E% carbohydrates but proteins originating from four different sources (dairy, fish, meat, and plant foods). The energy content was 700 kcal for the males' portions and 560 kcal for the females'. The content of Ile, Phe, and Tyr calculated using the amino acid food composition tables from the Swedish food administration [15] differed slightly between the four meals, with the highest amount of Ile and Tyr in the dairy meal and the highest amount of Phe in the plant foods meal (Table 2). A detailed description of the test meals and their total amino acid composition can be found in Supplementary Document  1 and Supplementary Table  1 (see Supplementary Material available online at http://dx.doi.org/10.1155/2016/8576730). The meals were consumed in the order: dairy, fish, plant, and meat, with a washout period of 7 days between the test days to ensure no bias caused by previous meal intake. Test meals were consumed between 8 and 8.30 a.m. together with 33 cl of noncarbonated water.

### 2.2. Blood Sample Collection

Blood samples for metabolic profiling were collected after a 10-hour overnight fast from 10 p.m. until 8 a.m. (*t* = 0) as well as 30, 60, 90, 120, and 180 minutes after test meal ingestion. Samples for analysis of blood glucose, triglycerides, HDL cholesterol, LDL cholesterol, total cholesterol, and insulin were collected in the overnight fasted state and at 120 minutes after completion of the meal. Measurements of height, weight, and waist circumference were conducted at the first visit for all participants.

### 2.3. Analytical Procedure

Metabolites were profiled in EDTA plasma using liquid chromatography-mass spectrometry (LC-MS) with a UHPLC-QTOF-MS System (Agilent Technologies 1290 LC, 6550 MS, Agilent Technologies, Santa Clara, CA, USA). Plasma samples stored at −80°C were thawed on ice before metabolite extraction was performed by adding 180 *µ*L extraction solvent (4°C) to 30 *µ*L plasma and incubate at 4°C with mixing at 1250 rpm during one hour. After incubation the samples were centrifuged for 20 minutes at 14000 ×g and the supernatants were transferred to glass vials. Extraction solvent consisted of 80 : 20 methanol/water (liquid chromatography grade) and the stable isotope labeled internal standards [16] phenylalanine-d8, isoleucine-d10, and tyrosine-d8 purchased from Cambridge Isotope Laboratories (Andover, MA, USA). Samples were separated on an Acquity UPLC BEH Amide column (1.7 *µ*m, 2.1*∗*100 mm; Waters Corporation, Milford, MA, USA) maintained at 40°C [17]. Solvent A is H_2_O, with 10 mM ammonium formate and 0.1% formic acid. Solvent B is acetonitrile with 0.1% formic acid. Gradient is as follows: 0–3 min, 100–95% B; 3–6 min, 95–80% B; 6–13 min, 80–70% B; 13-14 min, 70–40% B; 14–16 min, 40%; 16-17 min, 100% B. The flow rate was 0.4 mL/min and sample injection volume 2 *µ*L. The autosampler was kept at 16°C. Mass spectrometry was performed in positive electrospray ionization. The sheath gas temperature was set at 350°C and the sheath gas flow at 12 L/min. The drying gas flow was 14 L/min and was delivered at 200°C. Mass spectra were acquired at a rate of 1 spectrum/s and the mass range was 70–1000* m/z*. Samples were analyzed in batches of 45 samples, where pooled QC samples were injected every 5 samples and in the beginning of each batch to ensure high repeatability and conditioning of the LC-column, respectively [18].

### 2.4. Statistical Analysis

To investigate the individual's exposure to circulating amino acids, a postprandial amino acid response was defined as the 180-minute postprandial area under the curve (AUC) that was calculated using trapezoidal integration. Homeostatic model assessment of insulin resistance (HOMA-IR) was calculated as the fasting insulin concentration (mU/L) multiplied by the fasting glucose concentration (mmol/L) divided by 22.5. The fasting DMAA score was calculated as the sum of the overnight fasted metabolites concentration and the postprandial DMAA score as the area under the DMAA score postprandial profile. Differences in Ile, Phe, and Tyr mean plasma concentrations between the four test meals and differences in baseline characteristics of the participants were analyzed using one-way ANOVA. Tukey's honestly significant difference test (Tukey's HSD) was used to find specific differences between the diet groups. AUC calculation, one-way ANOVA, and Tukey's HSD were performed in R 3.2.0. The association between postprandial metabolite AUC and overnight fasting glucose was assessed using robust linear regression, clustered on participant ID, in order to account for repeated measurements. The analysis was performed using STATA. Associations between postprandial metabolite AUCs after intake of each test meal and fasting glucose and HOMA-IR were calculated with linear regression using SPSS 22. In all of the statistical analysis, *P* < 0.05 was considered as statistically significant.

## 3. Results

Characteristics of the participants included in the test meal protein challenge are shown in Table 1. There were no differences in baseline circulating levels of glucose, Ile, Phe, Tyr, triglycerides, LDL cholesterol, HDL cholesterol, total cholesterol, insulin, or HOMA-IR between any of the four different visits prior to the ingestion of the four meals (*P* > 0.05 for all comparisons).

### 3.1. The Postprandial Amino Acid Profile Is Affected by the Test Meal's Protein Source

**Figure 1:**
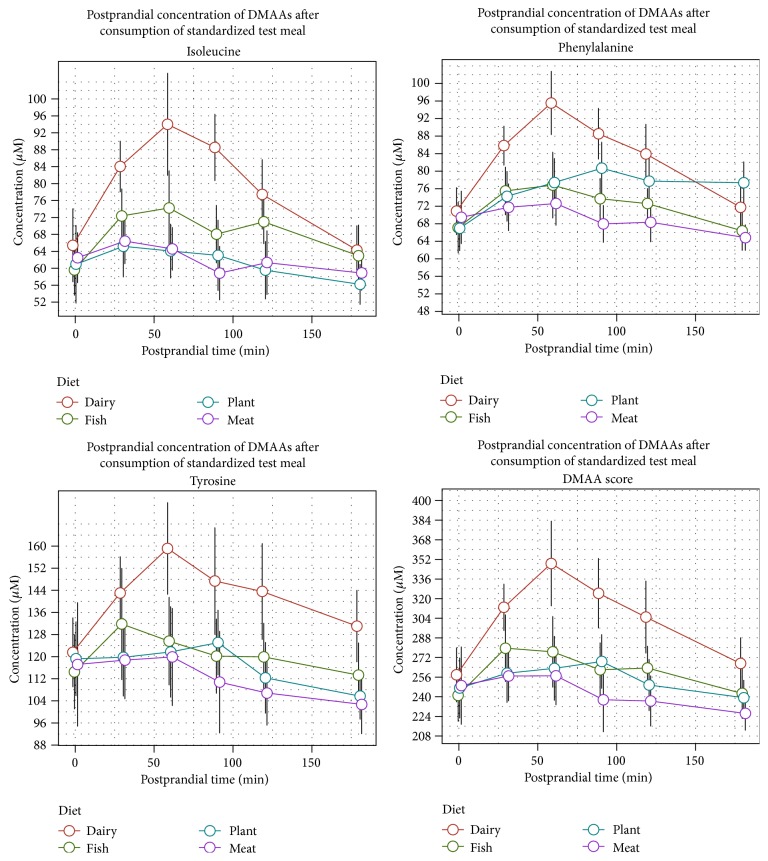
Postprandial amino acid profiles for isoleucine, phenylalanine, tyrosine, and DMAA score after consumption of test meals with four different protein sources.

Consumption of the dairy meal induced a distinct peak in plasma concentration for Ile, Phe, and Tyr 60 minutes after meal ingestion and triggered the highest postprandial concentrations for all the three amino acids. Consumption of the meals with fish, meat, or plant protein resulted in a low increase in postprandial DMAA concentrations and no distinct peak time of concentrations could be detected, indicating a slower appearance rate of these DMAAs in the circulation (Figure 1).

### 3.2. The Postprandial Amino Acid Response Was Increased after Ingestion of the Meal with Dairy Protein

**Figure 2:**
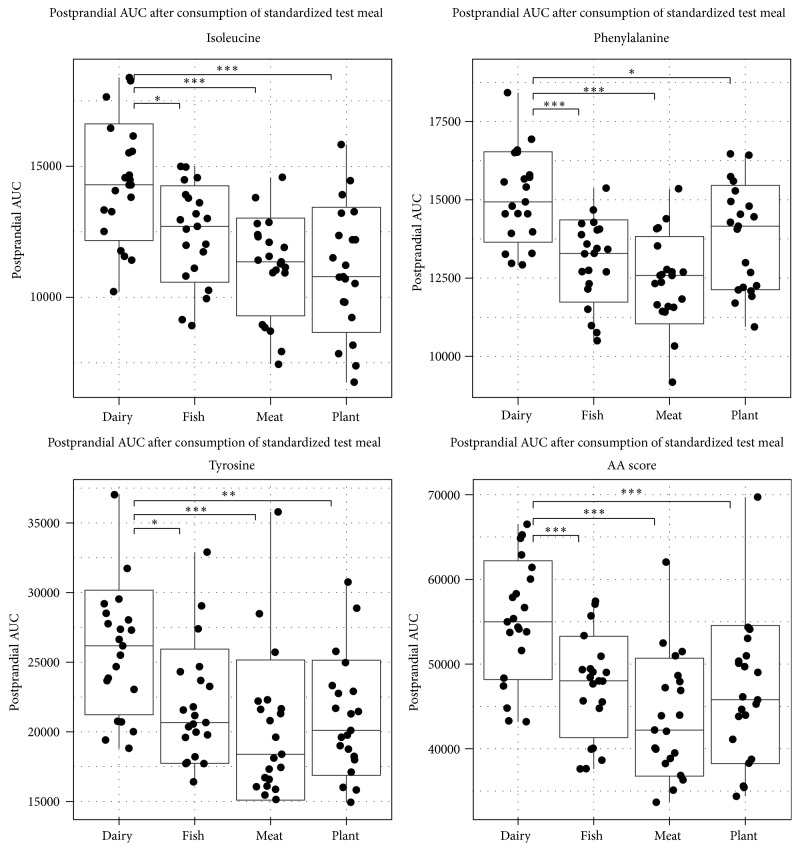
Boxplots of 180-minute postprandial AUC after intake of a test meal with proteins from four different sources. ^*∗*^ *P* < 0.05; ^*∗∗*^ *P* < 0.01; ^*∗∗∗*^ *P* < 0.001.

Differences in the postprandial amino acid AUC can be seen in Figure 2. The protein source in the test meal significantly affected the postprandial amino acid AUC for all the three amino acids and the amino acid score (*P* < 0.05), as presented in Supplementary Table  2. The dairy-based test meal resulted in a significantly greater postprandial amino acid AUC for the DMAA score and all the three individual amino acids in comparison to any of the three other test meals (*P* < 0.05 for all comparisons and metabolites) (Figure 2). The complete results from Tukey's HSD are presented in Supplementary Table  3.

### 3.3. Fasting and Postprandial Amino Acid Levels Associate with Fasting Glycaemia

Fasting DMAA score and Phe were associated with increased overnight fasting glycaemia (*P* = 0.034 and *P* = 0.045, resp.) while fasting Ile and Tyr showed no significant association. The postprandial AUC for all the three amino acids and the DMAA score was significantly associated with overnight fasting glycaemia and all associations were stronger than in the fasted state (Table 3). AUC of the DMAA score showed the strongest association (*P* = 0.006), followed by Phe (*P* = 0.008), Tyr (*P* = 0.013), and Ile (*P* = 0.015). All associations were adjusted for age and sex. Also, the postprandial AUCs were higher in overweight compared to normal weight participants: Ile: *P* = 0.02; Phe: *P* = 0.005; Tyr: *P* = 0.004; DMAA score: *P* = 0.001. Given the higher postprandial amino acid levels in overweight versus normal weight participants, BMI was used together with age and sex as a covariate in the linear regression analyses on fasting glucose; however the results were only marginally changed compared to age and sex adjusted analyses (Supplementary Table  4).

### 3.4. Postprandial Amino Acid Associations with Fasting Glycaemia after Intake of Specific Protein Sources

The postprandial AUCs of amino acids were significantly associated with fasting glycaemia after consumption of fish protein for Ile (*P* = 0.04) and Phe (*P* = 0.03), after plant protein for Phe (*P* = 0.05) and after meat protein for Tyr (*P* = 0.01) but not after consumption of dairy protein (the protein source which gave the greatest AUC for all individual amino acids) for any of the three amino acids. The postprandial DMAA score associated with fasting glycaemia after meat consumption (*P* = 0.027) and displayed borderline significant associations after ingestion of fish and plant protein (both associations* P* = 0.054), but no significant association was seen between the postprandial DMAA score and fasting glycaemia after dairy consumption. Detailed results of all diet specific associations are presented in Supplementary Table  5.

### 3.5. Fasting and Postprandial Amino Acid Levels Associate with Insulin Resistance

To describe the link between postprandial BCAAs and AAAs and the prediabetic phenotype, the relation between fasting and postprandial levels of the amino acids and insulin resistance, as measured with fasting HOMA-IR, was investigated (Table 4). Increased fasting DMAA score (*P* < 0.001), Tyr (*P* < 0.001), and Phe (*P* = 0.0053) associated with HOMA-IR, while fasting Ile (*P *= 0.41) did not. The postprandial level of Ile was significantly associated with fasting HOMA-IR (*P* = 0.004), while the AUC of Phe (*P* < 0.001) and DMAA score (*P* = 0.008) associated similarly with fasting HOMA-IR as their fasting metabolite measures. Postprandial levels of Tyr (*P* = 0.048) were also significantly associated with fasting HOMA-IR, although a weaker association was observed than for the fasting concentrations.

## 4. Discussion

The key findings of the study were that the postprandial AUCs of DMAAs were greater after consumption of a meal with dairy protein compared to plant, fish, and meat. Postprandial AUCs of DMAAs were more strongly associated with fasting glycaemia than the fasting concentrations of the DMAAs. Interestingly, the association between postprandial DMAAs and fasting glycaemia differed with the meal protein sources and was not seen when the dairy meal was consumed. This indicates that the DMAAs glycaemia association is washed out by the effect of dairy protein intake on the postprandial DMAAs levels. Levels of all DMAAs positively associated with HOMA-IR in both the fasted and the postprandial state except for Ile which showed no association in the fasted state.

Fasting concentrations of DMAAs have been shown to associate with glycaemia and insulin resistance and predict future diabetes. There is however great controversy about the mechanisms behind the associations and whether there is a causal relation between the metabolites and the clinical outcomes. Thus, currently there are no clues about whether the associations derive from amount of dietary protein intake, protein source in the diet, muscle protein breakdown, tissue uptake rate of the DMAAs, or altered metabolism of DMAAs. Identification of the responsible mechanism is the first step in order to understand whether controlling DMAA intake would be desirable in order to prevent diabetes or if manipulation of DMAAs has no effect. In this context, we believe that the postprandial kinetics and metabolism of the DMAAs have been largely overlooked and are in need of more examination. While most studies of postprandial amino acid levels are performed during an oral glucose tolerance test (OGTT) or investigate the effect of a specific treatment on postprandial amino acid levels, the main strength of the present study is that the amino acid profiles were obtained in healthy persons after ingestion of protein through a standardized, but still normal, meal. Thus we have the possibility of examining the postprandial kinetics of DMAAs in healthy subjects, how it is affected by the dietary protein source originating from a meal, and how fasting and postprandial DMAAs relate to fasting levels of glucose, the core phenotype defining diabetes mellitus.

### 4.1. Standardized Meal with Dairy Protein Increases Postprandial Amino Acid Response

We observed that the protein source of a test meal affected the postprandial levels of Ile, Phe, and Tyr. That all the three amino acids had significantly higher postprandial AUC after consuming dairy protein is unlikely to be solely explained by differences in the test meals amino acid content, as the dairy meal did not have consistently higher levels of all DMAAs. This indicates that there are additional mechanisms that contribute to this result. Intake of dairy proteins has previously been shown to result in increased postprandial DMAA and insulin levels compared to protein content-matched meals with fish and gluten as protein source. The more rapid increase in postprandial concentrations of DMAAs was suggested to be the result of a higher digestion and absorption rate of dairy proteins [19–21].

### 4.2. Postprandial Responses Show Stronger Association with Fasting Glycaemia than Fasting Concentrations

In the present study we show that overnight fasting circulating levels of Phe and of the DMAA score associated with fasting glycaemia, while the individual amino acids Ile and Tyr show no significant association. Previous studies demonstrating associations between fasting glucose and fasting levels of all of these amino acids have consisted of larger samples sizes [4, 6], which might explain why these results could not be reproduced in our study. The postprandial AUC of all the three individual amino acids and of the DMAA score associated with overnight fasting glycaemia. That stronger association with overnight fasting glycaemia that was found postprandially suggests that the connection between metabolic status, as estimated by overnight fasting glucose concentration, and DMAA levels is mainly related to events occurring in the postprandial state. Since muscle breakdown contributes minimally to changes of DMAAs postprandially, the altered DMAA levels should be explained either by the dietary intake, by the tissue uptake rate, or by differences in postprandial metabolism of DMAAs.

### 4.3. Postprandial Appearance and Clearance of DMAAs Have Been Associated with Metabolic Improvements

Three studies have shown that the improvement of risk factors for metabolic disorder, including insulin resistance, achieved through bariatric surgery was mirrored by the lowering of several plasma amino acids levels in both the fasted and postprandial state [14, 22, 23]. After surgery a more rapid DMAA clearance was observed and the postprandial insulin and amino acid profiles showed a stronger correlation. This indicates that the uptake of DMAAs in tissue is at least to some extent insulin dependent. In support, the postprandial uptake of Ile after an OGTT was shown to be significantly reduced in insulin resistant subjects [12]. The results from our study are concordant with this theory, since HOMA-IR was associated with the postprandial AUC of Ile but not with the fasting concentrations of Ile. On the other hand, this effect is not observed for Tyr and Phe, where the association with insulin resistance is either weaker or only marginally stronger for postprandial amino acid levels. Thus, one could speculate that different mechanisms are responsible for the association with insulin resistance for AAA compared to BCAA. In a recent study the increase in plasma leucine (Leu) concentration, a BCAA closely related to Ile, after protein ingestion was demonstrated to be significantly higher in elderly diabetics compared to age-matched normoglycaemic controls, further validating the connection between postprandial BCAAs and insulin status [24]. Another study investigating the uptake of circulating amino acids in muscle tissue could confirm different uptake rates between Leu and Phe. A negative net flux of Leu was observed in the postabsorptive state, while the influx to muscle was rapidly increased after protein ingestion, resulting in a positive net flux. The net flux of Phe was also changed after protein ingestion but the increase in inflow was much lower than for Leu [25]. Together with our results, these findings would suggest that the association between postprandial levels of BCAA (Ile) and fasting glucose reflects mainly insulin resistance, possibly mediated through muscle tissue, and the association between postprandial levels of AAA (Phe and Tyr) and fasting glucose might be driven partly by insulin resistance and partly by other mechanisms. The association between postprandial amino acid AUC and fasting glycaemia in our study is thus likely, at least partly, due to lower amino acids tissue uptake in individuals with higher fasting glucose. It is tempting to suggest that the decreased clearance of circulating DMAAs postprandially, as a result of impaired tissue uptake, might reflect the insulin resistant and diabetes prone phenotype.

### 4.4. No Association between Postprandial Amino Acid Response and Fasting Glycaemia after Dairy Consumption

There was no significant association between postprandial DMAA response and fasting glycaemia for any amino acid after consumption of dairy protein, which was the test meal that resulted in the greatest postprandial amino acids AUC. The effect of the dairy protein meal on postprandial DMAAs levels is likely explained by higher amino acid content and higher digestion and absorption rate of dairy proteins. The strong DMAA elevating effect of the dairy protein meal might wash out any association between postprandial DMAA AUC and fasting glycaemia.

## 5. Study Limitations

The present study has several limitations. A relatively small sample size of only 21 participants limits the conclusions that can be drawn from the study, suggesting the importance to replicate our findings in a larger sample size. Additionally, the order the meals were consumed was not randomized. Even though a washout period of seven days should ascertain that no bias was attributed to meal order, a randomized order would have been preferable.

## 6. Conclusions

The relation between postprandial AUC of DMAAs and fasting glycaemia and insulin resistance after a test meal reveals a connection between postprandial amino acid kinetics and metabolic status. Whether the postprandial levels of DMAAs are causally related to glycaemia and insulin resistance is beyond this study's scope but we suggest that a decreased tissue uptake rate might result in increased circulating postprandial DMAA levels in the diabetes prone state. Our study suggests that further investigation of postprandial amino acid levels might help shed light on the mechanisms behind the relation between DMAAs and glucose homeostasis.

## Supplementary Material

Supplementary material includes tables and charts of the calculated amino acid content in the four test meals (dairy, fish, plants and meat).

## Figures and Tables

**Table 1:** Clinical characteristics and overnight fasting metabolic measurements in 21 participants, with standard deviation before intake of four different protein challenge tests. *P* values are calculated using one-way ANOVA. Hyperglycaemia was defined as fasting glucose > 6.1 mM and overweight as BMI > 25.0.

	Dairy *n* = 21	Fish *n* = 21	Plant foods *n* = 21	Meat *n* = 21	*P*
Age (years)	34.5 (±9.9)				—
Sex (% female)	47.6				—
BMI (kg/m^2^)	24.8 (±2.8)				—
Waist (cm)	86.1 (±9.8)				
Overweight (%)	42.9				
Current smokers (%)	28.6				
Hyperglycaemia (%)	4.8				
Insulin (pM)	39.8 (±15)	41.1 (±18)	38.5 (±15)	37.8 (±15)	0.91
HOMA-IR	1.66 (±0.74)	1.69 (±0.93)	1.51 (±0.68)	1.49 (±0.70)	0.78
Glucose (mM)	5.56 (±0.63)	5.44 (±0.53)	5.23 (±0.51)	5.26 (±0.56)	0.19
Triglycerides (mM)	0.96 (±0.64)	0.85 (±0.34)	0.91 (±0.37)	1.01 (±0.39)	0.57
Cholesterol (mM)	4.54 (±0.58)	4.45 (±0.71)	4.64 (±0.72)	4.64 (±0.73)	0.79
LDL cholesterol (mM)	2.82 (±0.63)	2.75 (±0.65)	2.91 (±0.74)	2.83 (±0.71)	0.93
HDL cholesterol (mM)	1.62 (±0.39)	1.56 (±0.37)	1.64 (±0.41)	1.65 (±0.40)	0.80
Ile (*µ*M)	65.4 (±17.6)	59.6 (±12.9)	60.9 (±19.8)	62.5 (±12.8)	0.67
Phe (*µ*M)	70.9 (±11.6)	67.1 (±12.4)	67.0 (±11.1)	69.5 (±13.0)	0.62
Tyr (*µ*M)	121.6 (±26.9)	114.5 (±28.9)	129.5 (±62.0)	118.1 (±51.7)	0.73

**Table 2:** Amino acid content in four standardized test meals of 700 kcal for male participants. The amino acid content in the portions for female participants (560 kcal) consisted of 80% of each amino acid in each meal.

Metabolite	Dairy (mg)	Fish (mg)	Plant foods (mg)	Meat (mg)
Ile	1180	1088	911	1026
Phe	1084	888	1113	821
Tyr	1095	778	521	724
Total DMAA	3359	2754	2545	2571

**Table 3:** Robust linear regression of fasting blood glucose clustered on participant ID (*n* = 4*∗*21), adjusted for age and sex. Metabolites are overnight fasting plasma concentrations and 180-minute postprandial AUC in 21 individuals at four different occasions. Beta values reflect increment of mM glucose per standardized unit of DMAA. CI: confidence interval.

Metabolites	Beta (95% CI)	*P*	Beta (95% CI)	*P*
	Fasting concentrations		180 min postprandial AUC	
AA score	0.15 (0.01–0.28)	0.034	0.21 (0.07–0.36)	0.006
Phenylalanine	0.12 (0.00–0.30)	0.045	0.19 (0.06–0.33)	0.008
Tyrosine	0.12 (−0.02–0.25)	0.086	0.18 (0.04–0.32)	0.013
Isoleucine	0.05 (−0.08–0.19)	0.45	0.18 (0.04–0.32)	0.015

**Table 4:** Robust linear regression of HOMA-IR clustered on participant ID (*n* = 4*∗*21), adjusted for age and sex. Metabolites are overnight fasting plasma concentrations and 180-minute postprandial AUC in 21 individuals at four different occasions. Beta values reflect increment of HOMA-IR per standardized unit of DMAA. CI: confidence interval.

Metabolites	Beta (95% CI)	*P*	Beta (95% CI)	*P*
	Fasting concentrations		180 min postprandial AUC	
DMAA score	0.26 (0.13–0.39)	<0.001	0.14 (0.04–0.24)	0.008
Phenylalanine	0.26 (0.08–0.44)	0.0053	0.16 (0.08–0.25)	0.001
Tyrosine	0.24 (0.13–0.36)	<0.001	0.10 (0.01–0.20)	0.048
Isoleucine	0.06 (−0.08–0.19)	0.41	0.12 (0.04–0.20)	0.004
