# Supplementary material for: Postprandial Levels of Branch Chained and Aromatic Amino Acids Associate with Fasting Glycaemia
Source: J Amino Acids. 2016 May 5;2016:8576730. doi: 10.1155/2016/8576730 (PMC4871975; doi:10.1155/2016/8576730)
Supplement: Supplementary file 1 — Supplementary material includes tables and charts of the calculated amino acid content in the four test meals (dairy, fish, plants and meat). [file 8576730.f1.pdf]

Table 1. Food items in the four test meals (dairy, fish, plant and meat) for female subjects, with a total energy content of 560 kcal. Test meals for male subjects contained 125 % of the amount of each food item to reach a total energy content of 700 kcal.

Food Item	Dairy	Fish	Plant	Meat
	Amount (g)			
Margarine (80 % fat)	1	1	-	1
Corn starch	82	87	-	87
Red/yellow onion	12	12	12	12
Garlic	1	2	2	1
Tomatoes (canned)	106	76	155	116
Spinach (frozen)	20	20	20	20
Margarine (75% fat)	4	12	18	10
Chili peppers	2	2	2	2
Vegetable broth	10	10	10	10
Honey	3	3	3	3
White wine (1 % alcohol)	15	15	15	15
Water	0	40	100	0
Cheese (31 % fat)	18	-	-	-
Cottage cheese (4 % fat)	90	-	-	-
Milk (3 % fat)	50	-	-	-
Cod	-	65	-	-
Salmon	-	39	-	-
Lentils	-	-	24	-
Minced beef (10 % fat)	-	-	-	83

Table 2. One-way ANOVA of 180 minutes postprandial AUC between test-meals with four different sources of protein (dairy, fish, plant foods and meat) in 21 individuals.

Metabolites	F	P
**Phenylalanine**	12.9	6.0e-7
**Isoleucine**	11.6	2.2e-6
**AA Score**	10.2	9.3e-6
**Tyrosine**	6.4	6.1e-3

Table 3. Multiple comparisons of the postprandial AUC of DMAA Score, Isoleucine, Phenylalanine and Tyrosine after test meals with four different protein sources. Differences in means and p-values are calculated using Tukey´s HSD.

Comparison	DMAA Score	Isoleucine	Phenylalanine	Tyrosine
**Fish-Dairy**	-7883(0.0029)	-1977(0.016)	-2044(<0.001)	-3863(0.031)
**Meat-Dairy**	-11450(<0.001)	-3235(<0.001)	-2656(<0.001)	-5573(<0.001)
**Vegetable-Dairy**	-8779(<0.001)	-3345(<0.001)	-1295(0.026)	-4692(0.0054)
**Meat-Fish**	-3568(0.36)	-1258(0.22)	-612(0.53)	-1711(0.60)
**Vegetable-Fish**	-896(0.98)	-1368(0.16)	748(0.35)	-830(0.93)
**Vegetable-Meat**	-2673(0.61)	-110(0.99)	1361(0.018)	881(0.92)

Table 4. Robust linear regression of fasting blood glucose clustered on subject ID (n=4*21), adjusted for age, sex and BMI. Metabolites are overnight fasting plasma concentrations and 180 minutes postprandial AUC in 21 individuals at four different occasions. Beta values reflect increment of mM glucose per standardized unit of DMAA. CI, confidence interval.

Metabolites	Beta (95% C.I)	P	Beta (95% C.I)	P
	**Fasting Concentrations**		**180 min Postprandial AUC**	
**DMAA Score**	0.14(0.01-0.26)	0.041	0.22 (0.07-0.37)	0.004
**Phenylalanine**	0.17(0.02-0.32)	0.030	0.20(0.07-0.33)	0.003
**Tyrosine**	0.12(-0.03-0.27)	0.11	0.18(0.04-0.33)	0.014
**Isoleucine**	0.05(-0.10-0.20)	0.52	0.18 (0.04-0.32)	0.013

Table 5. Linear regressions of fasting glucose, adjusted for age and sex. Metabolites are 180 minutes postprandial AUC.

Metabolite	Dairy		Fish		Vegetable		Meat	
	Beta	P	Beta	P	Beta	P	Beta	P
**DMAA Score**	0.010	0.66	0.37	0.054	0.51	0.054	0.56	0.027
**Isoleucine**	-0.00	0.99	0.48	0.039	0.30	0.35	0.12	0.71
**Phenylalanine**	0.24	0.23	0.40	0.30	0.44	0.047	0.25	0.28
**Tyrosine**	0.06	0.79	0.26	0.19	0.39	0.13	0.66	0.01
